# Characterization of two bacterial multi-flavinylated proteins harboring multiple covalent flavin cofactors

**DOI:** 10.1016/j.bbadva.2023.100097

**Published:** 2023-07-05

**Authors:** Yapei Tong, Henriette J. Rozeboom, Marnix R. Loonstra, Hein J. Wijma, Marco W. Fraaije

**Affiliations:** Molecular Enzymology group, University of Groningen, Nijenborgh 4, Groningen 9747AG, the Netherlands

**Keywords:** Flavin transferase, Multi-flavinylated proteins, FMN

## Abstract

In recent years, studies have shown that a large number of bacteria secrete multi-flavinylated proteins. The exact roles and properties, of these extracellular flavoproteins that contain multiple covalently anchored FMN cofactors, are still largely unknown. Herein, we describe the biochemical and structural characterization of two multi-FMN-containing covalent flavoproteins, SaFMN3 from *Streptomyces azureus* and CbFMN4 from *Clostridiaceae bacterium*. Based on their primary structure, these proteins were predicted to contain three and four covalently tethered FMN cofactors, respectively. The genes encoding SaFMN3 and CbFMN4 were heterologously coexpressed with a flavin transferase (ApbE) in *Escherichia coli*, and could be purified by affinity chromatography in good yields. Both proteins were found to be soluble and to contain covalently bound FMN molecules. The SaFMN3 protein was studied in more detail and found to display a single redox potential (-184 mV) while harboring three covalently attached flavins. This is in line with the high sequence similarity when the domains of each flavoprotein are compared. The fully reduced form of SaFMN3 is able to use dioxygen as electron acceptor. Single domains from both proteins were expressed, purified and crystallized. The crystal structures were elucidated, which confirmed that the flavin cofactor is covalently attached to a threonine. Comparison of both crystal structures revealed a high similarity, even in the flavin binding pocket. Based on the crystal structure, mutants of the SaFMN3-D2 domain were designed to improve its fluorescence quantum yield by changing the microenvironment of the isoalloxazine moiety of the flavin cofactor. Residues that quench the flavin fluorescence were successfully identified. Our study reveals biochemical details of multi-FMN-containing proteins, contributing to a better understanding of their role in bacteria and providing leads to future utilization of these flavoprotein in biotechnology.

## Highlights

• First detailed characterization of multi-FMN-containing proteins.
• Two bacterial flavoproteins, containing 3 and 4 covalent flavin cofactors, respectively, could be produced and purified as recombinant proteins.
• The fluorescence and redox properties have been determined.
• The crystal structures of two single FMN-containing domain have been elucidated.
• Mutant proteins were prepared with altered fluorescence properties.
• Reduced FMN-containing protein can consume molecular oxygen.

## Graphical abstract

**Figure:**
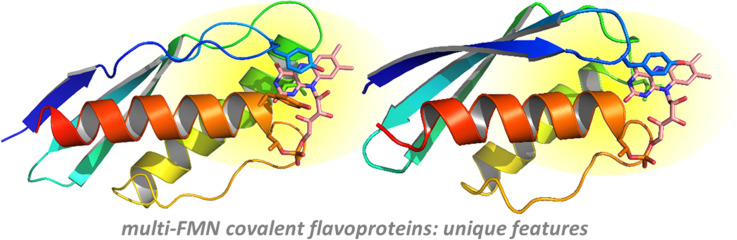

## Introduction

Flavins are essential cofactors required for metabolic processes within all living organisms [1,2]. Flavin mononucleotide (FMN) and flavin adenine dinucleotide (FAD) are the canonical flavin cofactors [2], [3], [4]. Because of the redox-active isoalloxazine ring, flavin cofactors are capable of existing in either fully oxidized, one-electron reduced, or two-electron reduced states. This allows flavoproteins to catalyze a wide variety of biochemical redox reactions [5,6]. Most flavoproteins bind their cofactor tightly through multiple noncovalent protein-flavin interactions. Still, about 10% of all flavoproteins contain a covalently attached FAD or FMN. In most cases, such flavin-protein linkage is formed via the isoalloxazine group and typically involves a histidine, tyrosine, aspartate, or cysteine residue [7]. A totally different type of covalent flavin-protein bond was first identified in the subunits of the Na^+^-translocating NADH:quinone oxidoreductase (Na^+^-NQR) from *Vibrio alginolyticus* [8,9]. In this membrane-bound multimeric flavoenzyme, the phosphate group of FMN forms a phosphoester bond with a threonine residue in the conserved DGxSGAT sequence motif. A dedicated flavin transferase, called ApbE, catalyzes this post-translational FMN-protein conjugation reaction, using FAD as precursor [10].

Recent studies have revealed that many bacterial and archaeal genomes contain ApbE homologs and multiple genes encoding proteins with one or more Dxx[S/T]GA[S/T]-like sequence motifs [11]. Specifically, ApbE-flavinylated FMN-binding domains have been found in many (predicted) extracytosolic electron transfer systems [12], [13], [14], [15], [16], including Na^+^-NQR [17], rhodobacter nitrogen fixation (RNF) [12,18], nitrous oxide reduction [15], organohalide reduction [13], and extracellular electron transfer [14]. Recently, Méheust et al. discovered by sequence analysis a multitude of sequences of extracytosolic proteins predicted to contain multiple covalently bound FMN molecules [11]. Such multi-FMN binding proteins are thought to play a role in extracellular long-distance electron transfer paths similar to multi-heme proteins [19,20]. The exact roles and properties of these widespread multi-flavinylated proteins in bacteria have remained unclear. In this study, we present the biochemical characterization of two predicted multi-flavinylated proteins, SaFMN3 and CbFMN4, from *Streptomyces azureus* and *Clostridiaceae bacterium*, respectively. The sequence of SaFMN3 harbors three putative FMN-binding motifs, which suggests that it is recognized by a flavin transferase for covalent coupling of three flavin cofactors in each SaFMN3 molecule. Likewise, CbFMN4 is predicted to bind four FMN molecules. The genes coding for these two multi-flavin proteins are flanked by genes for putative flavin transferases, which further supports that they bind FMN covalently.

In this investigation, by coexpressing these two proteins with AbpE, we indeed managed to obtain multi-flavinylated proteins. Also truncated versions of SaFMN3 and CbFMN4, comprising the sequence of one of their domains, were obtained and studied. The results provide first insights into the biochemical properties of these multi-flavin proteins and may help to understand their role in extracellular processes. These covalent flavoproteins may also develop into tools for biotechnology as they display interesting and tunable redox and fluorescence properties.

## Results and discussion

### Properties of recombinant proteins

Through a bioinformatic analysis, Méheust et al. identified a large number of bacterial proteins that are predicted to contain one or more covalent FMNs [11]. The number of covalently bound FMNs in one protein varied from 1 to 13. Although a few multi-flavinylated proteins have been studied in detail [14,17], knowledge on extracellular multi-flavinylated FMN-binding proteins is extremely limited. In this investigation, we expressed and studied two multi-FMN-binding proteins, SaFMN3 and CbFMN4, that were predicted to carry three and four covalent FMNs, respectively. A BLAST search (NCBI) with the SaFMN3 and CbFMN4 protein sequence confirmed that these two proteins belong to the FMN-binding protein superfamily in view of its highly conserved FMN binding motifs (DxxxGAT). Protein sequence analysis by using the RADAR (Rapid Automatic Detection and Alignment of Repeats in protein sequences tool, https://www.ebi.ac.uk/Tools/pfa/radar/) showed that both proteins harbor several highly homologous domains [21]. A previous study suggests that such organization of three/four long repeat sequences may hint to an important role in the structure and function of the protein [22]. TMHMM (TransMembrane prediction using Hidden Markov Models, https://dtu.biolib.com/DeepTMHMM) [23] analysis predicted that both proteins have a secretion signal and a transmembrane sequence at their N-terminus [24]. This is in line with the extracellular membrane-bound localization of flavin transferases in bacteria. Apparently, these multi-FMN-containing proteins are anchored on the outside of the cells.

**Fig. 1:**
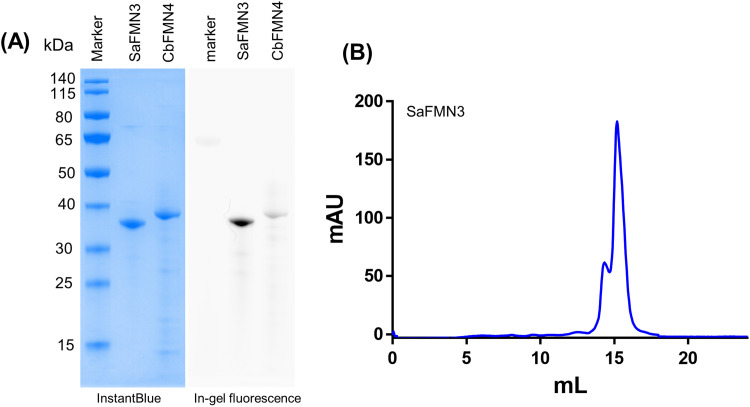
Purification of SaFMN3. (A) Polyacrylamide gel of purified SaFMN3 and CbFMN4. In-gel fluorescence shown at the right while the same gel after protein-staining is at the left. Lane 1, marker; lane 2, flavinylated SaFMN3; lane 3, CbFMN4. (B) Gel permeation chromatography profile for SUMO-tag-cleaved SaFMN3.

Both proteins were expressed as His-tagged SUMO fusion proteins, and co-expressed with ApbE and CaFADS in *E. coli* BL21-AI. Expression of ApbE, a flavin transferase, is needed for the covalent tethering of the FMN cofactors to the recombinant proteins using FAD as precursor, while CaFADS ensures sufficient FAD synthesis in the recombinant cells [25]. The N-termini (residues 1–48 for SaFMN3 and residues 1–26 for CbFMN4) were truncated during the cloning procedure, resulting in high yields of intracellular and soluble protein. Both proteins could be expressed at high levels without extensive expression optimization (∼120 mg from 1 L of TB medium). Upon removal of the His-tagged SUMO by incubating with SUMO protease, untagged SaFMN3 and CbFMN4 were obtained as brightly yellow protein solutions. SDS−PAGE analysis showed a protein band of about 35 kDa, slightly higher when compared with the theoretical molecular mass of the produced SaFMN3 (theoretical mass of flavinylated SaFMN3 = 31.1 kDa, apo SaFMN3 = 29.8 kDa) (Fig. 1A). The protein band was also clearly fluorescent suggesting that it contains covalently attached flavin (Fig. 1A). The native molecular mass of SaFMN3 was determined by size-exclusion chromatography. The protein eluted in a major peak at 15 mL, corresponding to a monomeric protein of 30 kDa, while a preceding shoulder appears to correspond to the dimeric species (Fig. 1B). Thus, the elution profile indicates that in solution SaFMN3 mainly exists as a monomer. The thermal stability of SaFMN3 was investigated by measuring the apparent melting temperature (T_m_) using the ThermoFAD method [26]. This method relies on a change in microenvironment of the flavin cofactor, indicative of an unfolding of the protein structure. SaFMN3 was found to have an apparent T_m_ value of 48 °C.

For purified CbFMN4, SDS-PAGE revealed a protein band corresponding to a somewhat lower molecular mass (38 kDa) when compared with the predicted mass (theoretical mass of flavinylated SaFMN4 = 44.8 kDa, apo SaFMN4 = 43.0 kDa) (Fig. 1A). Unfortunately, ESI-MS analysis of this protein was found to be problematic. Despite testing several settings and conditions, no proper MS data could be obtained. For SaFMN3, MS analysis was successful (vide infra). Hence, we decided to focus on analysis of the flavinylated SaFMN3.

### Spectral characteristics of flavinylated SaFMN3

**Fig. 2:**
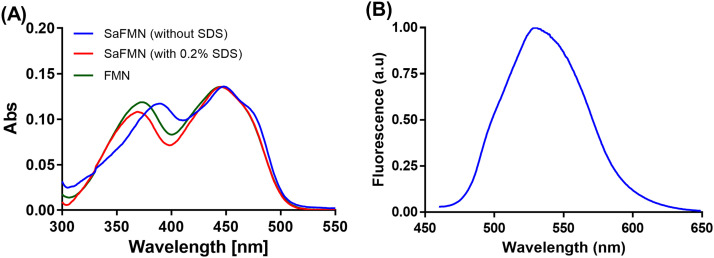
UV-visible absorption and emission spectra of SaFMN3. (A) UV-visible absorption spectra of purified SaFMN3 in native state (blue) and upon unfolding by 0.2% SDS (red), and free FMN as reference (dark green), (B) Emission spectrum of SaFMN3 obtained by exciting the sample at a wavelength of 448 nm and measuring the fluorescence emission in a range of 460−650 nm.

The purified SaFMN3 displayed an intense yellow color in solution, indicating that FMN was bound to the protein. SaFMN3 in the oxidized form displayed the typical UV–visible absorbance spectrum of FMN-containing flavoproteins, with two absorbance maxima in the visible region at 450 and 375 nm (Fig. 2A). When SaFMN3 was denatured with 0.2% SDS, a slightly altered spectrum was obtained due to unfolding of the protein, resembling FMN in solution (Fig. 2A). TCA precipitation of the protein resulted in a yellow protein pellet which again confirms that it contained covalently attached FMN. In order to study whether SaFMN3 incorporated three FMN molecules, occupying all three predicted FMN-binding motifs, we carried out ESI-MS analysis using SUMO-tag free samples to determine the molecular mass of SaFMN3. The ESI-MS detected mass was 31,100 Da which exactly corresponds to the predicted mass of fully flavinylated SaFMN3 (Figure S1A).

SaFMN3, with three FMN molecules bound in a 30 kDa protein, may display interesting fluorescence properties. It may be an attractive fluorescent protein for labeling studies. Therefore, the fluorescence quantum yield of SaFMN3 was determined. The fluorescence was monitored at an excitation wavelength of 448 nm where it has an absorbance maximum (Fig. 2A). The emission spectra of purified samples were recorded from 460 nm to 650 nm (Fig. 2B). As described in previous studies [27], we used fluorescein as reference (known quantum yield, Φ_f_, of 0.91) to characterize the fluorescence quantum yield of SaFMN3 and FMN (as a control). The quantum yield of free FMN (Φ_f_ = 0.20) measured in this work is indeed in agreement with previously determined values [28]. SaFMN3 showed an extremely low fluorescence quantum yield of 0.006.

### Crystal structures of SaFMN3-D2 and CbFMN4-D1

**Fig. 3:**
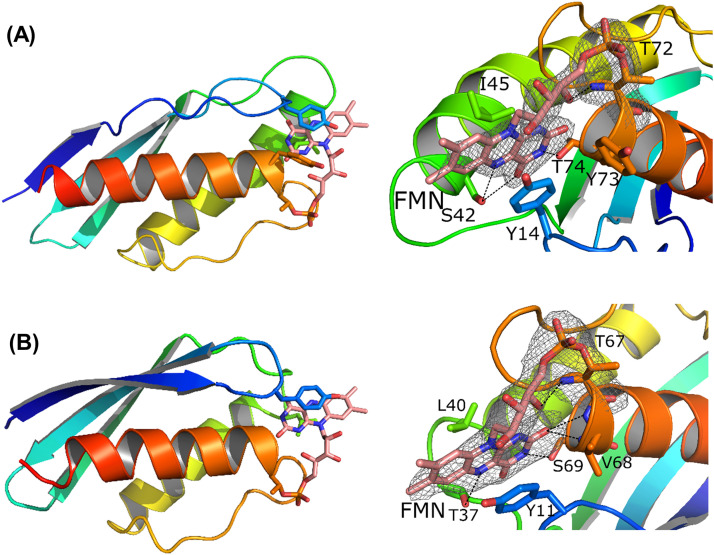
The crystal structures of SaFMN3-D2 and CbFMN4-D1. (A) Cartoon representation and Fo-Fc electron density map of SaFMN3-D2. (B) Cartoon representation and Fo-Fc electron density map of CbFMN4-D1. The N-terminus is shown in blue, the C-terminus in red, and FMN is shown with salmon sticks. Electron density of the covalently bound FMN and the linking threonine is shown in gray mesh, contoured at 2.5 σ level for SaFMN3-D2 and 3.0 σ for CbFMN4-D1.

To have a better view of the structural details of SaFMN3 and CbFMN4, we set out to determine their three-dimensional structures. Attempts to crystallize the full proteins failed, presumably due to a high flexibility of the three/four separate FMN-binding domains. The domains are separated by linker sequences that are rich in small residues, suggesting a high degree of flexibility (Figure S2, Table S1). An AlphaFold2-generated model confirms that the individual domains are separated by non-structured linkers with low pLDDT scores (predicted local distance difference test scores), as has been observed for unstructured regions before (Figure S2) [29]. As the sequences of the domains in each protein were highly similar, with sequence identities of >80%, we decided to determine the structure of one of the domains for each protein. The second domain of SaFMN3 (SaFMN3-D2, residues 157–244) and the first domain of CbFMN4 (CbFMN4-D1, residues 26–109) could be overexpressed as soluble proteins. Both proteins were yellow after purification and fully flavinylated as revealed by ESI-MS analysis. Both FMN-containing proteins could be crystallized, allowing elucidation of their crystal structures (Fig. 3).

The SaFMN3-D2 (9.4 kDa) structural organization consists of three β-strands forming an anti-parallel sheet (residues 2–6, 18–24 and 27–35), followed by two α-helices (residues 41–60 and 72–87) connected by a loop. The molecule has dimensions of 26 × 26 × 44 Å. An unusual position of Pro51, in the middle of the first helix, only leads to a minor distortion of this helix. The FMN-binding site is located between the N-terminal parts of the first helix and second helix. Most prominent is the hydrophobic stacking interaction of Tyr14 on the *si*-face of the isoalloxazine and Ile45 on its *re*-face (Fig. 3A). Furthermore, hydrogen bonds are located between the O2 of FMN and the backbone amides of Tyr73 and Thr74, the N3 with the Thr74-Oγ1, and the N5 with the Oγ of Ser42. The xylene ring is facing the solvent. The O4’ of the ribityl group makes a hydrogen bond to the backbone amide of Thr72. The O2P oxygen of the phosphate group has hydrogen bonding interactions with the backbone amide of Gly70. The phosphate group of the FMN is clearly covalently attached to the Oγ1 of Thr72 (Fig. 3A), which is located at the start of the last α-helix.

The asymmetric unit of the CbFMN4-D1 crystal contains two protein molecules. One domain displays electron density for residues 1–83, the other for residues 1–81. The CbFMN4-D1 structure consists, like SaFMN3-D2, of an anti-parallel sheet (residues 1–9, 14–22, and 25–33) and two α-helices (residues 38–56 and 67–83) (Fig. 3B). Hydrogen bonds are observed between the N-terminal strands of the two molecules. The dimer interface appears to have resulted from crystal packing. Tyr11 stacks on one site of the isoalloxazine (analogous to Tyr14 in SaFMN3-D2), while Leu40 is situated on the other site (analogous to Ile45 in SaFMN3-D2). Additionally, mostly analogous hydrogen bonds are observed between the O2 of FMN and the backbone amides of Val68 and Ser69, the N3 with Ser69-Oγ1, the O4 with the backbone amide of Thr37, and N5 with the Oγ1 of Thr37, while the O4’ of the ribityl group has a hydrogen bond with the backbone amide of Thr67. Both CbFMN4-D1 molecules have a covalently bound FMN via Thr67.

The structure of CbFMN4-D1 is similar to SaFMN3-D2 (their Cα r.m.s.d is 1.7 Å) while there is only 18% sequence identity. The highest similarity is observed in the FMN binding sequence motif. SaFMN3-D2 has, however, a small loop (GGR) inserted at the end of the third strand. The closest structural homologue of SaFMN3-D2 and CbFMN4-D1 is the CPE2226 protein from *Clostridium perfringens* (PDB:3O6U, not published). CPE2226 is also annotated as an “FMN bind domain-containing protein”. SaFMN3-D2 has a Cα r.m.s.d. of 1.7 Å for 79 aligned residues with 28% sequence identity to CPE2226 (CbFMN4-D1 r.m.s.d 2.4 Å, 23% identity). A more detailed comparison was performed between CPE2226 and SaFMN3-D2, the closest homolog. SaFMN3-D2 is missing the C-terminal β-strand of CPE2226. Furthermore, SaFMN3-D2 has a short loop of 7 residues after the last strand and before the first helix which in CPE2226 is substituted by a short excursion and an extra α-helix (total of 24 residues). The position of the covalent flavin-binding threonine is conserved in CPE2226 (Thr119). However, Tyr14 is changed to His44 in CPE2226, which does not have stacking interactions with its potential FMN binding site. The tip of the loop on which this histidine resides has shifted 6 Å away from the binding site. Possible this loop will move toward the site upon binding of FMN. The CPE2226 crystal structure does not contain an FMN cofactor, presumably because it was expressed in the absence of a flavin transferase. Hydrophobic interaction of CPE2226 on the other side of FMN could be provided by Met85. Moreover, Pro51 in the middle of the first helix of SaFMN3-D2 is conserved in CPE2226.

### SaFMN3-D2 variants with improved fluorescence quantum yield

The SaFMN3-D2 displayed a fluorescent quantum yield of 0.02, which is somewhat higher when compared with full length SaFMN3 but still about 10 times lower than that of free FMN (Table 1). The structure of SaFMN3-D2 shows that FMN closely interacts with the side chains of Tyr14, Ile45, and Tyr73. Especially the benzylic moiety of the isoalloxazine is sandwiched between Ile45 and Tyr14, which may lead to quenching of fluorescence (Fig. 3B). To gain a better insight into the effects of these three amino acids on the fluorescence properties of SaFMN3-D2, the Tyr14Ala, Ile45Ala, and Tyr73Ala mutants were prepared and analyzed for their fluorescent quantum yield (Table 1). Before determining the quantum yields of these mutants, their absorption spectra were measured. This revealed that all SaFMN3-D2 variants had very similar absorbance spectra with maxima at 447−450 nm. The fluorescence quantum yield of the three SaFMN3-D2 mutants exhibited a slightly increase when compared to SaFMN3-D2 (0.03–0.05 vs 0.02). Next, several double and triple SaFMN3-D2 mutants were prepared. It is worth noting that all generated mutants did contain covalently incorporated FMN. It shows that folding and posttranslational flavinylation was unperturbed with all probed single, double and triple mutants. The highest fluorescence quantum yield among all mutants was 0.07 for SaFMN3-D2-Tyr14Ala/Tyr73Ala and SaFMN3-D2-Tyr14Ala/Tyr73Ala/Ile45Ala. These data suggest that the Tyr14 and Tyr73 side chains have a fluorescence quenching effect on the bound FMN.

**Table 1:** Fluorescent quantum yield of SaFMN3 and its variants. The fluorescent quantum yields were measured in triplicate.

FMN/proteins	fluorescence quantum yield
FMN	0.20 ± 0.04
full length SaFMN3	0.006 ± 0.01
SaFMN3-D2	0.02 ± 0.01
SaFMN3-D2-Tyr14Ala	0.05 ± 0.02
SaFMN3-D2-Ile45Ala	0.04 ± 0.01
SaFMN3-D2-Tyr73Ala	0.03 ± 0.01
SaFMN3-D2-Tyr14Ala/Ile45Ala	0.05 ± 0.03
SaFMN3-D2-Tyr14Ala/Tyr73Ala	0.07 ± 0.02
SaFMN3-D2-Tyr73Ala/Ile45Ala	0.03 ± 0.01
SaFMN3-D2-Tyr14Ala/Tyr73Ala/Ile45Ala	0.07 ± 0.01

### Redox properties of SaFMN3

**Fig. 4:**
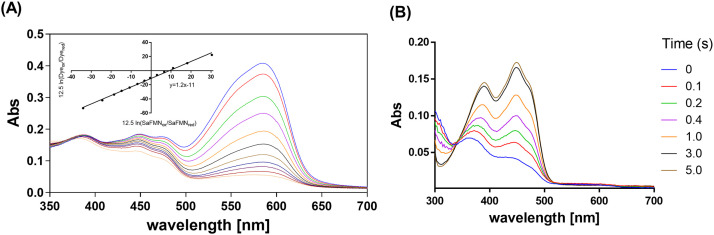
Redox properties of SaFMN3. (A) Redox titration of SaFMN3 with cresyl violet as the reporter dye (E_0_ = −166 mV). The decreases in absorbance of the FMN cofactor and cresyl violet could be followed at 450 and 585 nm, respectively. The inset shows a Nernst plot, in which the factor 12.5 (= *R*T/*nF*, in which *R* is gas constant, T the absolute temperature, *n* the number of transferred electrons, and *F* the Faraday constant) converts the concentrations of the redox active species to the solution potential relative to their own midpoint potential [31]. The thus determined redox potential of SaFMN3, obtained by comparison to the dye, is -184 ± 10 mV (two independent measurements). Measurements were performed in 50 mM potassium phosphate, pH 7.0, at 25 °C. (B) Reoxidation of dithionite-reduced SaFMN3 was monitored using a stopped-flow instrument. Reduced SaFMN3 (15 μM) was mixed with oxygen (final concentration 130 μM), in 50 mM potassium phosphate, pH 7.0, at 25 °C.

In most covalent flavoproteins, the covalent bond links the flavin isoalloxazine ring via an amino acid side chain [7]. This type of linkage often results in a drastic change in the flavin redox potential [2]. By contrast, in SaFMN3, the flavin cofactor is linked via a threonyl phosphoester bond which is not expected to have a direct effect on the redox properties of the isoalloxazine moiety. Yet, the protein environment around the redox active part of the flavin can still influence its midpoint potential. To explore the redox properties of SaFMN3, the flavin redox potential was determined. The midpoint potential of SaFMN3 was determined at pH 7.0 using the xanthine oxidase/dye equilibration method described by Massey, which is detailed in the Materials and Methods section [30], [31], [32]. The full reduction of SaFMN3 was monitored and revealed no spectral intermediate formation indicative of a one-electron-reduced species or significant differences between the three FMN cofactors. Using cresyl violet as reference dye, the redox potential of SaFMN3 was found to be −184 mV (Fig. 4A), which is slightly higher than that of free FMN (−207 mV).

### Oxygen reactivity of SaFMN3

We investigated whether the multi-flavinylated protein SaFMN3 can react with dioxygen when it is in its fully reduced state. Flavoproteins are known for their ability to generate reduced oxygen species, hydrogen peroxide and superoxide [33], by using molecular oxygen as electron acceptor. The reduced flavoprotein was prepared by titrating it with sodium dithionite under anaerobic conditions. Subsequently, a stopped-flow instrument was used to mix the reduced SaFMN3 with aerated buffer (0.26 mM oxygen). Upon mixing with aerated buffer, the absorbance spectrum of the oxidized cofactor promptly reappeared. Clearly, the reduced form of SaFMN3 reacts with dioxygen resulting in the oxidized enzyme. The reaction proceeded via a single-step mechanism, without appearance of any reaction intermediates, with a rate of 1.2 *s* ^−^ ^1^ (Fig. 4B). This suggests that SaFMN3 may have a catalytic role in scavenging oxygen and/or generating hydrogen peroxide and superoxide. Unfortunately, as the SaFMN3 protein could not be used as catalyst in a continuous mode, the amounts of reduced oxygen species were too small to determine the amounts of both reduced oxygen species. Yet, it has been shown that flavoproteins typically mainly generate hydrogen peroxide, while only small amounts of superoxide are formed.

## Conclusions

In the present work, we experimentally demonstrate that SaFMN3, lacking the predicted N-terminal membrane anchor, is a soluble monomeric multi-flavinylated protein when coexpressed with ApbE. The protein contains three FMN molecules covalently tethered to three threonyl residues *via* a phosphoester bond. The protein is comprised of three highly homologous independent domains. The crystal structure of one of the domains, SaFMN3-D2, was elucidated and revealed details of the flavin-binding pocket. Except for confirmation of the predicted site of covalent FMN attachment, three residues were identified that encompass the isoalloxazine moiety of the FMN cofactor, possibly contributing to the observed fluorescence quenching of the flavin. Mutagenesis revealed that two tyrosines have the largest quenching effect. It shows that protein engineering can be used to tune the fluorescence properties of such multi-FMN proteins or of individual FMN-decorated domains.

Another multi-FMN-containing protein was also expressed and studied: CbFMN4. Structural elucidation of a domain of this flavoprotein revealed a similar overall structure and binding mode of the FMN cofactor. Clearly, these covalent multidomain flavoproteins are widespread among bacteria, displaying a similar structure for each flavoprotein domain, suggesting a similar role.

Our results reveal some interesting features of these multi-flavin proteins that may hint to a physiological role. A redox titration of SaFMN3 resulted in a single and relatively high redox potential (−184 mV versus the normal hydrogen electrode). Apparently, all three bound FMNs have a similar redox behavior. This is also in line with the high sequence identities (84–94% sequence identity) when the three domains are compared. The structural model also suggests that the domains are connected by relatively long flexible loops. It suggests that there is no directionality for a potential redox transfer process via the bound flavins.

Interestingly, we have found that the reduced form of SaFMN3 readily reacts with molecular oxygen via a two-electron transfer process, yielding fully oxidized SaFMN3. As such, it acts as an oxidase, generating reduced oxygen species. Such activity can also help to scavenge dioxygen. The natural electron donor for such activity is unknown. When inspecting the crystal structure of the SaFMN3-D2, no clear binding pocket next to the isoalloxazine moiety can be identified that could bind an organic substrate. Possibly, the flavins are reduced via single electron transfers from other protein partners or chemical processes. An activity as oxidase may be relevant to lower oxygen concentrations or to produce hydrogen peroxide for combatting competing organisms or assisting on some (bio)chemical processes. Future research on SaFMN3 or sequence-related FMN-containing protein will reveal the exact role of these extracellular covalently attached flavins.

Except for a better understanding of their role, further studies on these proteins may yield useful molecular tools. We have shown that the fluorescence properties can be tuned by protein engineering. The individual domains of only 9 kDa may be developed as fusion tags, allowing easy detection of fusion proteins based on their color and fluorescence.

## Materials and methods

### Chemicals, and strains

Ni-Sepharose 6 Fast Flow was from Cytiva. Mutagenic primers were also ordered from Sigma-Aldrich (Merck; St. Louis, MO, USA). T4 ligase was purchased from ThermoFisher and the restriction enzyme BsaI was purchased from New England Biolabs. The *E. coli* NEB 10-beta and BL21-AI (NEB, Ipswich, MA, USA) strains were used as hosts for cloning and protein expression, respectively. All other reagents and chemicals were purchased from Sigma-Aldrich.

### Cloning, transformation, and mutagenesis

The gene sequence encoding SaFMN3 (WP_059422874.1) from *Streptomyces azureus* and CbFMN4 (PWFQ01000014.1/18,527–19,831) from *Clostridiaceae bacterium* isolate T1Sed10_28 T1-Sed10-C80 were synthesized by Twist Bioscience. The *safmn3* and *cbfmn4* genes with BsaI sites at the 5′ and 3′ termini were cloned into a pBAD-His6x-SUMO (small ubiquitin-like modifier) vector by using the Golden Gate cloning approach [34]. The standard Golden Gate reaction mixture contained the pBAD-His6x-SUMO vector, BsaI restriction enzyme, T4 ligase, ligation buffer, the gene, and sterile Milli-Q water. The incubation temperature first alternated for 30 cycles between 37 °C for 5 min and 16 °C for 10 min, was then set to 55 °C for 10 min, and finally to 65 °C for 20 min to inactivate the enzymes. Five μL of this reaction mixture was added to chemically competent *E. coli* NEB 10-beta cells for transformation and subsequent plasmid production. After overnight growth on an LB agar plate with ampicillin, colonies were picked and grown in LB medium with ampicillin. The plasmids were isolated and sent for sequencing (GATC, Germany) to confirm the correct ligation of the genes. All the protein sequences used for this work are shown in Table S1. The pRSF-Duet-1 vector, which carries a flavin transferase gene and a FAD synthetase gene, was used for coexpression with the multi-flavinylated protein [25]. The *E. coli* BL21-AI strain was used for protein expression.

Site-directed mutagenesis was carried out by using the pBAD-NHis-SUMO-SaFMN3-D2 vector as a template. The used primers are listed in Table S2. For PCR, the 20 μL reaction mixtures contained 30 ng template, 0.2 μM (each) mixed primers and 10 μL PfuUltra II Hotstart PCR Master Mix. The latter contained optimized PCR reaction buffer, magnesium, and dNTPs. The 20 μL mixture was subjected to the following: first 95 °C for 2 min, then 30 cycles of 95 °C for 20 s, 55 °C for 30 s, 72 °C for 2 min, followed by a final extension at 72 °C for 10 min. The PCR products were digested with DpnI at 37 °C to remove the parental templates, after which the reaction mixtures were transformed into chemically competent *E. coli* NEB 10-beta. Plasmids were isolated and sent for sequencing to confirm the mutations.

### Expression and purification

To express flavinylated proteins, the pBAD-His6x-SUMO vectors with the *safmn3* or *cbfmn4* gene and the pRSF-Duet-1 vector with flavin transferase and FAD synthetase genes were transformed into *E. coli* BL21-AI cells. CbFMN4, SaFMN3 and its variants were coexpressed with ApbE and CaFADS at 30 °C for 24 h in 200 mL TB medium containing 50 μg/mL ampicillin, 50 μg mL^−1^ kanamycin, and 100 mg/L riboflavin. l-arabinose (0.02% w/v) and IPTG (0.5 mM) were added when the OD_600_ was around 0.8 – 1.0. Cell pellets was resuspended in 50 mM Tris–HCl buffer at pH 8, containing 100 mM MgCl_2_, and 1 μg/mL DNase. Cells were disrupted by sonication (3 s on, 4 s off, 70% amplitude for a total of 10 min) and spun down by centrifugation at 12,000 rpm for 30 min. The His6x-tagged proteins were purified using 4 mL HisTrap Ni-Sepharose HP columns (GE Healthcare Lifesciences, USA) and desalted with a HiPrep 26/10 Desalting column (Cytiva), using 50 mM Tris–HCl buffer pH 8. Purified protein was stored at −70 °C for further use. The purified protein concentration was determined by measuring the absorbance at 450 nm and using an extinction coefficient of 13,112 *M* ^−^ ^1^ cm^−1^ for SaFMN3 and 11,816 *M* ^−^ ^1^cm^−1^ for SaFMN3-D2. The molar absorption coefficient was determined after unfolding in 0.2% SDS, and comparison with the known FMN absorption spectrum [35,36]. The samples were diluted to 1.0 µM before carrying out the mass spectrometry experiment for the covalent FMN binding analysis.

### Size exclusion chromatography

SUMO was cleaved from SUMO fused SaFMN3 by adding SUMO protease at 4 °C overnight. Then recombinant proteins without SUMO (5 −10 mg/mL) were used for gel filtration analysis on an ÄKTA purifier (GE Healthcare Lifesciences, USA) using a Superdex200 10/300 GL column (Cytiva), equilibrated with 50 mM Tris–HCl (pH 8.0) buffer containing 200 mm NaCl. Thyroglobulin (670,000 Da), γ-globulin (158,000 Da), ovalbumin (44,000 Da), myoglobin (17,000 Da), and vitamin B12 (1350 Da) were used as reference molecules to estimate the apparent molecular mass.

### ESI-MS analysis

Electrospray ionization mass spectrometry (ESI-MS) was used to verify the FMN incorporation and determine the protein mass. Samples were applied to ESI-MS using the Waters® Xevo® G2 Tof/ACQUITY UPLC H—Class® System (Waters) coupled to a quadrupole/time-of-flight (QToF) mass spectrometer (Waters) equipped with a PDA detector. The method employed was similar as in previous work [25,37]. Protein samples were diluted to 1 μM and 4 μL samples were injected. The protein samples were first separated on an Acquity BEH C4 (150 × 2.1 mm, 1.7 μm, Waters) column operated at 40 °C. Protein were eluted using a gradient from solvent A (0.1% formic acid in water) to B (0.1% formic acid in acetonitrile) at a flow rate of 0.3 mL/ min using the following protocol: 2 min at 95% A, 13 min to go from 95% to 5% A. Mass spectra were obtained in the ESI-positive ion mode. The obtained charge density spectra were deconvoluted using the MagTran software.

### Thermostability assays

To determine the thermostability of the studied proteins, the apparent melting temperature was measured by using the ThermoFAD method [26]. The samples (20 μL) contained 20 μM purified protein in 50 mM Tris–HCl buffer at pH 8.0. The measurements were performed using a RT-PCR thermocycler (CFX96 from Bio-Rad). The temperature was increased from 25 °C to 95 °C at a rate of 0.5 °C/min. The maximum of the first derivative of the observed flavin fluorescence change was taken as the apparent melting temperature.

### TCA protein precipitation

TCA (trichloroacetic acid) protein precipitation experiments were carried out to confirm that the cofactor was covalently bind to SaFMN3 and CbFMN4. Briefly, 100% (w/v) TCA was added to the protein samples to a final concentration of 5%, after which these samples were kept on ice for 1 h, followed by centrifugation for 10 min [38].

### Fluorescence quantum yield analysis

For fluorescence quantum yield (Φ_f_) measurements, proteins and fluorescein (as a reference standard) were diluted in 1x Phosphate-buffered saline pH 7.4 (137 mM NaCl, 2.7 mM KCl, 10 mM Na_2_HPO_4_, 1.8 mM KH_2_PO_4_) and 0.10 M NaOH buffer, respectively. Fluorescence spectra of SaFMN3 were recorded by exciting the sample at an excitation wavelength of 448 nm using a JASCO FP-8300 spectrofluorometer and measuring the fluorescence emission in the range of 460−650 nm. The integrated fluorescence intensity was calculated from the fluorescence spectra based on the formula $\Phi_{f\;}^{x}=\;\Phi_{f\;}^{R}\left(\frac{F_{x}}{F_{R}}\right)\left(\frac{f_{R}}{f_{x}}\right)\left(\frac{n_{x}^{2}}{n_{R}^{2}}\right)$, where subscript X denotes the fluorescent species which is newly analyzed, subscript R the reference fluorescent species, *F* the integrated intensity (areas), *f* is an absorption factor (*e.g. f_x_* = 1 – 10^−^*^ax^*, where *A* = absorbance <0.1), *n* is the refractive index of the solvent (NaOH for standard reference, and 1x PBS for sample), and $\Phi_{f\;}^{R}$ is the quantum yield of the reference fluorophore fluorescein ($\Phi_{f\;}^{R}$ = 0.91). [27,39]

### Redox potential determination

The redox titration of SaFMN3 was performed spectroscopically according to the xanthine/xanthine oxidase electron delivering system as described by Massey [30]. The reaction was performed in 50 mM potassium phosphate, pH 7.0, at 25 °C, containing 5.0 μM benzyl viologen as a mediator, 50 mM glucose, 5.0 μg/mL catalase, 50 μg/mL glucose oxidase, 400 μM xanthine, and xanthine oxidase in a catalytic amount [30,31,40]. The glucose/glucose oxidase is used to remove oxygen from the reaction mixture [41]. Catalase is added to the reaction solution to remove the hydrogen peroxide produced by glucose oxidase. Anaerobic conditions of the reactions were established by 10 min flushing with argon. The spectra were collected over a period of 60 min. The dye cresyl violet (*E*_0_ = −166 mV) was used as reference dye for redox potential determination of SaFMN3. The *E*_M_ values were determined using the Nernst equation. Reduction was initiated by adding xanthine oxidase and subsequently monitored at 25 °C using a spectrophotometer (V-330, JASCO) equipped with a thermostatic cell compartment. Spectra (200 – 700 nm) were recorded every 80 s until complete reduction of dye and flavoprotein.

### Reoxidation of SaFMN3

The reoxidation of reduced SaFMN3 was studied using the single-mixing mode of an SX20 stopped-flow spectrophotometer equipped with a photodiode array detector (Applied Photophysics, Surrey, UK). All solutions were prepared in 50 mM Tris–HCl, pH 8.0 buffer. Reactions performed by mixing equal volumes of two solutions at 25 °C. The stopped-flow instrument was made anaerobic by flushing the flow-circuit with a dioxygen-scrubbing solution containing 5.0 mM glucose and 0.30 μM glucose oxidase (*Aspergillus niger*, type VII, Sigma-Aldrich). To prepare fully reduced SaFMN3, titrations of oxidized proteins with sodium dithionite were carried out in a vial under anaerobic conditions. After adding a small aliquot (< 10 μL) of sodium dithionite to the protein (15 μM), the protein was completely reduced, immediately. The reduced flavoprotein was loaded into the stopped-flow instrument and mixed with air-saturated buffer (0.26 mM dioxygen) to monitor the spectral changes taking place in the stopped-flow cell. All data were analyzed using the software Pro-kineticist (Applied Photophysics, Surrey, UK).

### Crystal structures of SaFMN3-D2 and CbFMN4-D1

SUMO cleaved SaFMN3-D2 and CbFMN4-D1 were further purified by gel permeation chromatography at 280 K using a Superdex75 10/300 GL column (Cytiva), equilibrated with 20 mM Tris buffer, pH 7.5, containing 150 mM NaCl. The absorbance was monitored at 273, 382, and 450 nm. Fractions were pooled and concentrated to ∼15 mg mL^−1^ using an Amicon Ultra centrifugal filter unit (Merck Millipore Ltd., USA) with a 3 kDa cut-off.

Initial sitting-drop crystallization screening was performed using a Mosquito crystallization robot (STP Labtech) in 96-well MRC2 plates. Several crystallization screens were tested at 294 K. Almost all crystallization drops stayed clear for both proteins. After several weeks of incubation, a hole was punched, with a preparation needle, in the sealing sheet at the reservoir position and experiments were left to evaporate. After one week, tiny yellow SaFMN3-D2 crystals were grown from 20% w/v PEG 3350 at pH 6.5 – 7.0. Crystallization conditions were further optimized with the Dragonfly (STP Labtech). Final crystals were grown using 38 to 42% PEG 3350 and 0.05 M Bis-tris pH 7.0 as reservoir solution. The best FMN4-D1 crystals could be obtained from 3.4 M ammonium sulfate and 0.1 M bis-tris propane pH 7.7.

Prior to data collection, crystals were briefly soaked in a cryoprotectant solution containing 42% w/v PEG 3350, and 0.05 M Bis-tris pH 7.0, and flash-cooled in liquid nitrogen. As cryoprotectant for CbFMN4-D1 crystals, 3.4 M ammonium sulfate and 15% glycerol was used. X-ray diffraction data were collected at ESRF beamline MASSIF-1 [42]. Intensity data were integrated with XDS [43] and scaled with the AIMLESS routine [44] from the CCP4 software suite [45]. To generate a search model, Alphafold 2.1 [46] was used with standard settings and using known protein structures with release dates up to the 10th of December of 2021. Molecular replacement was performed with PHASER [47].

The resulting structures were improved by several rounds of model building and refinement, using the programs Coot [48] and REFMAC5 [49], alternately. The FMN was built in the binding site in Fo – Fc electron density map and covalently attached to the proteins. TLS rigid body refinement [50] was used in the last steps of refinement. The quality of the models was analyzed with PDB_REDO [51] and MolProbity. [52] PyMOL was used for structure analysis and figure preparation. Data collection statistics and refinement details are listed in Table 2. Atomic coordinates and experimental structure factor amplitudes were deposited in the Protein Data Bank PDB number 8P2A for SaFMN3-D2 and PDB number 8P2B for CbFMN4-D1.

**Table 2:** Data collection and refinement statistics. Numbers in parenthesis are for the highest resolution shell.

*Data collection*	SaFMN3-D2	CbFMN4-D1
Beamline	ESRF/MASSIF-1	ESRF/MASSIF-1
Wavelength (Å)	0.9655	0.9655
Resolution range (Å)	45.6 - 2.00 (2.05- 2.00)	40.9 – 2.60 (2.72–2.60)
Space group	P6_5_22	P6_3_
V_M_ Å^3^ Da−1, solvent (%)	1.9, 35	3.4, 63
Molecules per asymmetric unit	1	2
Unit cell dimensions a,b,c, (Å)	26.0, 26.0, 364.8	124.9, 124.9, 29.9
CC_(1/2)_	0.9967 (0.982)	0.998 (0.526)
<I/σ>	9.0 (2.4)	13.7 (0.8)
R_merge_	0.139 (0.441)	0.154 (2.449)
R_pim_	0.039 (0.137)	0.052 (0.894)
Completeness (%)	100(100.0)	100.0 (100.0)
Multiplicity	13.1 (11.0)	9.7 (9.5)
*Refinement*		
Atoms in A.U. protein/solvent/FMN	627 / 23 / 30	1261 / 12 / 60
Average B-factors, protein/solvent/FMN (Å^2^)	22.4 /43.9 / 34.6	62.6 / 53.8 / 44.7
R / R_free_ (%)	23.1 / 29.8	18.2 / 21.9
Bond lengths rmsd (Å)	0.008	0.008
Bond angles rmsd (°)	1.51	1.38
Ramachandran preferred/outliers (%)	97.7 / 0.0	95.0 / 0.6
Rotamers preferred/outliers (%)	90.5 / 3.2	89.1 / 2.9
Clashscore (percentile)	9.13 (86th)	4.35 (99th)
Molprobity score (percentile)	1.93 (79th)	1.91 (98th)
PDB accession code	8P2A	8P2B

## Declaration of Competing Interest

The authors declare the following financial interests/personal relationships which may be considered as potential competing interests:

Yapei Tong reports financial support was provided by CSC - Chinese scholarship Council.

## Data Availability

Data will be made available on request.

## Appendix Supplementary materials

Image, application 1
