# Supplementary material for: Characterization of two bacterial multi-flavinylated proteins harboring multiple covalent flavin cofactors
Source: BBA Adv. 2023 Jul 5;4:100097. doi: 10.1016/j.bbadva.2023.100097 (PMC10339131; doi:10.1016/j.bbadva.2023.100097)
Supplement: Supplementary file 1 [file mmc1.docx]

**Supplementary Information**

Characterization of two bacterial multi-flavinylated proteins harboring multiple covalent flavin cofactors

Yapei Tong, Henriette J. Rozeboom, Marnix R. Loonstra, Hein J. Wijma, Marco W. Fraaije*

Molecular Enzymology group, University of Groningen, Nijenborgh 4, 9747AG, Groningen, The Netherlands

* corresponding author: M.W. Fraaije

E-mail: m.w.fraaije@rug.nl

**Figure S1**


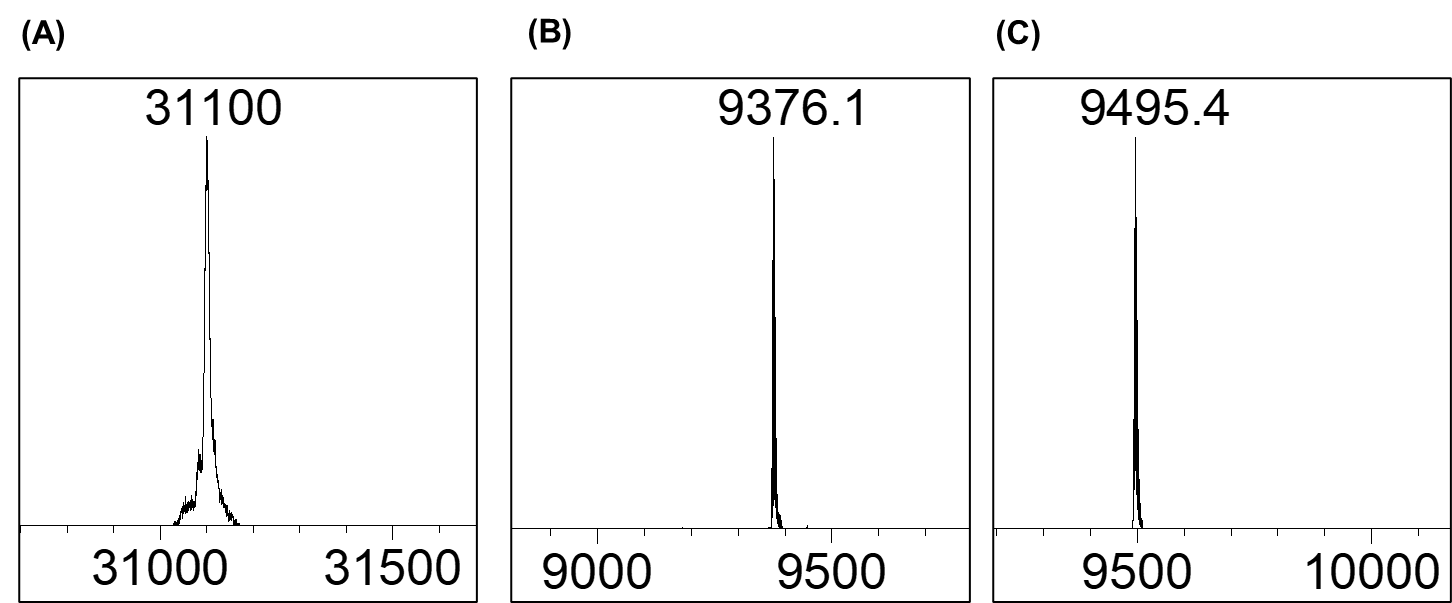


**Figure S1**. **Electrospray ionization mass analysis of full length SaFMN3, SaFMN3-D2 and CbFMN4-D1.** All proteins were purified after coexpression in the presence of ApbE and CaFADS. (A) The mass corresponds to the mass of fully flavinylated SaFMN3 (31,100 Da). (B) The mass corresponds to the mass of flavinylated SaFMN3-D2 (9,376 Da). (C) The mass corresponds to the mass of flavinylated SaFMN4-D1 (9,495 Da).

**Figure S2**

**
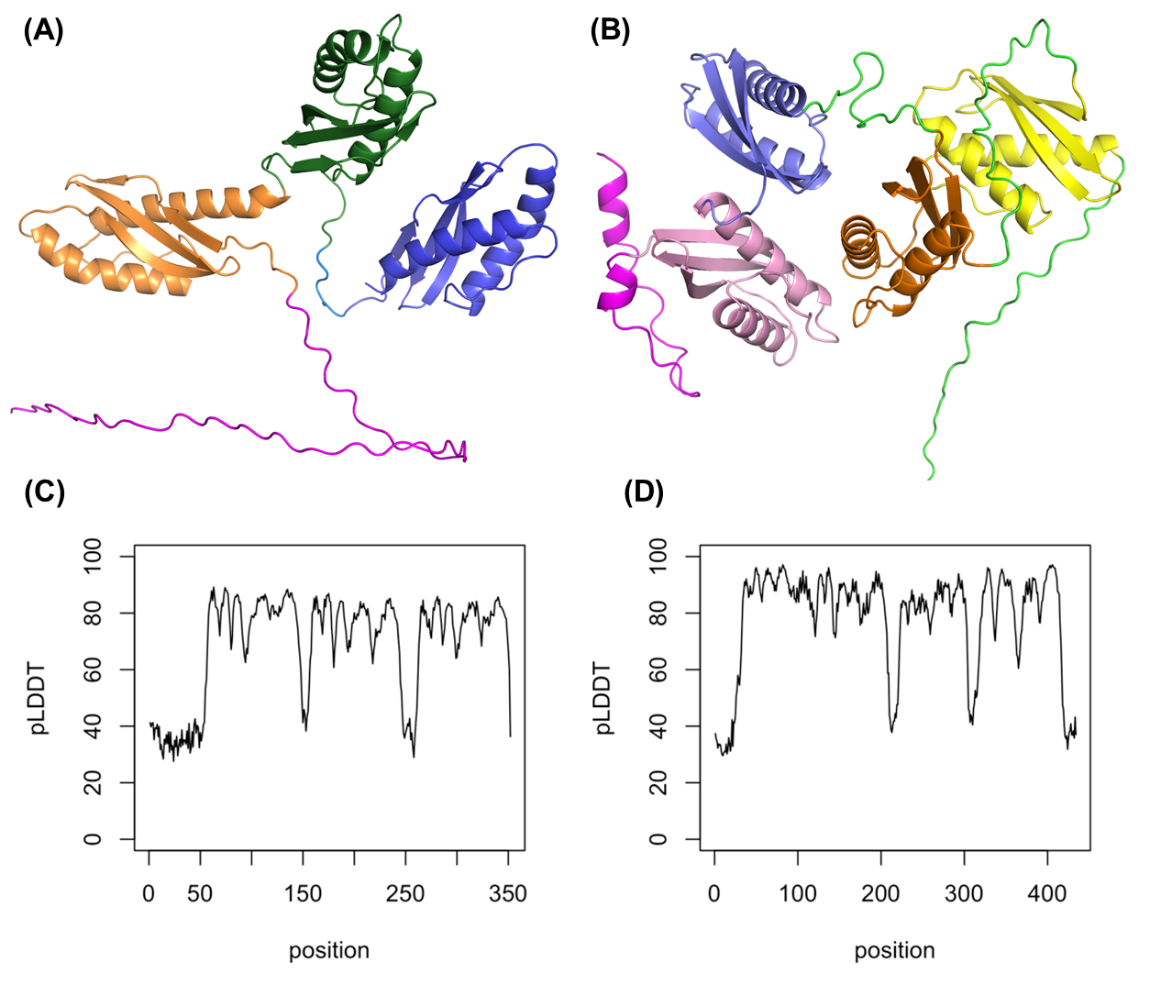
**

**Figure S2. Predicted structures of SaFMN3 and CbFMN4.** The three-dimensional structure of SaFMN3 and CbFMN4 were modeled using Alphafold2. (A) Predicted structure of SaFMN3, the transmembrane part is in magenta, the SaFMN3-domain 1, domain 2, and domain 3 were shown in orange, green, and blue, respectively. (B) Predicted structure of CbFMN4, the transmembrane part is in magenta, the CbFMN4-domain 1, domain 2, domain 3, and domain 4 were shown in pink, blue, orange, and yellow, respectively. (C) plot of predicted local distance difference test scores as calculated by Alphafold2 for SaFMN3. (D) pLDDT scores for CbFMN4.

**Table S1. Protein sequences used in this study.**

| **protein** | **amino acid sequence** |
| --- | --- |
| SaFMN3 | M**RKSHPVRRAVLAGAATVSGIVLLLSLKPASDPGSAQAAGGQFPPAAA**GQAPQGGVNGAVTGDAAQTQYGAVQVRLTMSGGKITQAEAVQAPKGGRSDQITASSVPRLNQAAVAAQSADIDAVSGATYTSAGYKKSLQSALDKAKASAGGAQGSGNAQTVTGDVAQTQYGPVQVRITVAGGKITKAEAVQAPKGGRSDQITSASVPRLNQAAVAAGSAEIDAVSGATYTSAGYKKSLQSALDKAPAGGGSSQAAGSGGGQAQTRTLTGSVAQTQYGPVQVRITVAGGKITKAEAVQAPKGGRSDQITSASVPRLNQAAVAAGNAQIDAVSGATYTSAGYKQSLQSALDQAGG |
| SaFMN3-D2 | MAQTVTGDVAQTQYGPVQVRITVAGGKITKAEAVQAPKGGRSDQITSASVPRLNQAAVAAGSAEIDAVSGATYTSAGYKKSLQSALDKA |
| CbFMN4 | M**QKKQIIALLTMVVLAVGLLFGYEALS**DDVLADALSYEVTAEGYGGPIRLMVYVEGEEIVDIEVLEENETPNLGDVAIEEMITKILEGQSTDVDVHSGATVSSNAVIEAVKQAMAEAGLVSGDAYVGVGAGYGGDVEVEVILDGNAIVAINVISENETADLGDVAIDEMIEKIIENQSVDVDVRSGATVSSNALIEAVKNALEEAGITLGEDDESSIEFDAEGILAIAPGYGGDIVLDIIMDGEEILEIRVLDQNETGGIGDIAIDEMIEKIIEAQSTDVDVQSGATVSSEAVIKAVAEAMGQEASASEPPQDPSADYDLETYEPEGIMVSGKGFQDRYDIHLDVIFDGNQIVEIRVIEHNETTGFGDGALRVVTERIINQQNTDVDIQTGATWTSNSTMELVQQAVEEADIVLEEQEVSEDATSTDDGGGGGG |
| CbFMN4-D1 | MLSYEVTAEGYGGPIRLMVYVEGEEIVDIEVLEENETPNLGDVAIEEMITKILEGQSTDVDVHSGATVSSNAVIEAVKQAMAEAG |
| ApbE | MEKPAEQVHLSGPTMGTTYNIKYIQQPGIADSKILQTEIDRLLEEVNDQMSTYRKDSELSRFNQHTSSEPFAVSTQTLTVVKEAIRLNGLTEGALDVTVGPLVNLWGFGPEARPDVVPTDEELNARRAITGIEHLTIEGNTLSKDIPELYVDLSTIAKGWGVDVVADYLQSQGIENYMVEIGGEIRLKGLNRDGVPWRIAIEKPSVDQRSVQEIIEPGDYAIATSGDYRNYFEQDGVRYSHIIDPTTGRPINNRVVSVTVLDKSCMTADGLATGLMVMGEERGMAVAEANQIPVLMIVKTDDGFKEYASSSFKPFLSK |
| CaFADs | MDIWYGTAAVPKDLDNSAVTIGVFDGVHRGHQKLINATVEKAREVGAKAIMVTFDPHPVSVFLPRRAPLGITTLAERFALAESFGIDGVLVIDFTRELSGTSPEKYVEFLLEDTLHASHVVVGANFTFGENAAGTADSLRQICQSRLTVDVIDLLDDEGVRISSTTVREFLSEGDVARANWALGRHFYVTGPVVRGAGRGGKELGFPTANQYFHDTVALPADGVYAGWLTILPTEAPVSGNMEPEVAYAAAISVGTNPTFGDEQRSVESFVLDRDADLYGHDVKVEFVDHVRAMEKFDSVEQLLEVMAKDVQKTRTLLAQDVQAHKMAPETYFLQAES |

The flavinylation motifs are shown in red. Predicted domains are shown in grey. The N-terminal parts that have been truncated are shown in bold. N-termini of SaFMN3 (1-48 residues) and CbFMN4 (1- 26 residues) were truncated during the cloning in order to obtain soluble protein.

**Table S2. Primer sequences used in this study.**

| primers | primer sequence |
| --- | --- |
| SaFMN3-D2-Fw | GAGGTCTCGTGGTATGGCGCAAACAGTTACTGGTGAT |
| SaFMN3-D2-Rv | GTGGTCTCGCAAGTTACGCCTTGTCTAAGGCGCTTTG |
| SaFMN4-D1-Fw | GAGGTCTCGTGGTATGCTGAGCTATGAAGT |
| SaFMN4-D1-Rv | GTGGTCTCGCAAGTTATCCTGCTTCGGCCAT |
| SaFMN3-D2-Tyr14Ala-Fw | GTTGCACAAACACAGGCGGGACCCGTTCAG |
| SaFMN3-D2-Tyr14Ala-Fw | CTGAACGGGTCCCGCCTGTGTTTGTGCAAC |
| SaFMN3-D2-Ile45Ala-Fw | GACGTAGTGATCAAGCGACAAGTGCAT |
| SaFMN3-D2-Ile45Ala-Rv | ATGCACTTGTCGCTTGATCACTACGTC |
| SaFMN3-D2-Tyr73Ala-Fw | TCAGGCGCGACAGCGACTTCAGCTGGTTAC |
| SaFMN3-D2-Try73Ala-Rv | GTAACCAGCTGAAGTCGCTGTCGCGCCTGA |
